# Using a Direct Observation Tool (TOC-CEX) to Standardize Transitions of Care by Residents at a Community Hospital

**DOI:** 10.31486/toj.20.0154

**Published:** 2021

**Authors:** Heidi Kenaga, Tsveti Markova, R. Brent Stansfield, Tess McCready, Sarwan Kumar

**Affiliations:** ^1^ Wayne State University School of Medicine Office of Graduate Medical Education, Detroit, MI; ^2^ Medical University of South Carolina, Charleston, SC; ^3^ Wayne State University School of Medicine, Detroit, MI; ^4^ Transitional Year and Family Medicine Residency Programs, Ascension Providence Rochester Hospital, Rochester, MI; ^5^ Internal Medicine Residency Program, Ascension Providence Rochester Hospital, Rochester, MI

**Keywords:** *Direct observation instrument*, *hospitals—teaching*, *internship and residency*, *patient handoff*, *patient transfer*

## Abstract

**Background:** High-quality transitions of care are crucial for patient safety in hospitals, yet few undergraduate curricula include transition-of-care training. In 2012, the Wayne State University Office of Graduate Medical Education (WSUGME) required its residency programs to use the SAIF-IR mnemonic (summary, active issues, if-then contingency planning, follow-up activities, interactive questioning, readback) to ensure accurate and uniform handoffs. Subsequent program evaluations indicated that resident awareness and adoption of the mnemonic at our primary clinical site, Ascension Providence Rochester Hospital (APRH), could be improved. According to our institution's 2016 Clinical Learning Environment Review (CLER), 88% of residents reported following a standardized transition of care handoff, and 53% reported that faculty rarely supervised their handoffs. A 2016 WSUGME internal survey also revealed low rates of awareness (7% to 10%) of the mandated mnemonic. WSUGME then created a direct observation tool, the Transitions of Care-Clinical Evaluation Exercise (TOC-CEX), for faculty to monitor resident skill in using the mnemonic and thus standardize transitions of care as a practice habit at APRH.

**Methods:** Since 2014, WSUGME had relied on 2 methods for training residents in the required handoff mnemonic: (1) introduction to the SAIF-IR mnemonic during the WSUGME orientation for all interns and (2) simulations during an objective simulated handoff evaluation activity for all postgraduate year (PGY) 1s and PGY 2s. In 2017, WSUGME innovated a direct observation tool, the TOC-CEX, for adoption by faculty at APRH to assess resident knowledge of and monitor their skill in using the SAIF-IR mnemonic in 3 primary care programs. The total number of possible participants was 138, and the actual number of individuals in the sample was 95. A majority (86%) of the observations during the study period were of PGY 1 residents, and thus the analysis reflects the ratings of 99% of all interns but only 69% of all possible residents.

**Results:** WSUGME found that faculty use of a direct observation instrument in the clinical learning environment during 2017-2019 increased awareness and adoption of the SAIF-IR mnemonic among residents. Using a *z*-test of equal proportions on resident responses on an internal WSUGME survey, we found a significant rise in the percentage reporting yes to the question “Does your program have a mechanism for monitoring handoffs?” (*χ^2^*[3]=23.6, *P*<0.0001) and in the percentage identifying SAIF-IR in response to the question “Does your program endorse a specific mnemonic for organizing the contents of a verbal handoff?” (*χ^2^*[3]=45.0, *P*<0.0001). The increase from 2016 to 2017 is the result of the implementation of the TOC-CEX in the interim (question 1: *χ^2^*[1]=12.4, *P*<0.0005; question 2: *χ^2^*[1]=10.1, *P*<0.0025).

**Conclusion:** Our research found that use of the TOC-CEX to monitor resident handoffs resulted in improved awareness and adoption of the SAIF-IR mnemonic in the clinical learning environment. Program leadership reported that the practice was both feasible and well accepted by residents, faculty, and the APRH chief medical officer as the TOC-CEX became a customary component of APRH organizational culture and was perceived as central to quality patient care.

## INTRODUCTION

High-quality transitions of care (TOCs) are crucial for patient safety and quality of care in hospitals.^1,2^ Poor communication during TOCs has long been recognized as a leading cause of medical errors.^3-5^ Yet few medical schools include TOC training as part of their curriculum, and as a result, residency programs are challenged to provide effective handoff education and evaluate trainee skill in doing handoffs.^6,7^ In 2010, the Accreditation Council for Graduate Medical Education (ACGME) mandated that residency programs adopt a TOC policy to reduce medical errors and better ensure patient safety; monitoring handoffs is a central component of this policy.^8,9^

In 2012, the Wayne State University Office of Graduate Medical Education (WSUGME) established a task force to explore TOC protocols and selected the SAIF-IR mnemonic (summary, active issues, if-then contingency planning, follow-up activities, interactive questioning, readback) to ensure uniform and accurate handoffs.^10^ In fall 2013, WSUGME launched a pilot study to test resident application of the SAIF-IR mnemonic and developed a training regimen that included didactic sessions, objective simulated handoff evaluation activities at clinical sites or the school of medicine's clinical skills simulation center, and pre/post survey evaluation measures. That study, published in 2015, showed increases in residents’ awareness of the importance of accurate and thorough communication during handoffs but relied on self-reported data with no documentation of TOC use in the clinical setting.^11^

WSUGME's subsequent examination of data sources verifying the extent of residents’ adoption of the mnemonic was inconclusive, indicating that the desired goal of standardizing the use of the SAIF-IR mnemonic had not been fully achieved. According to our institution's May 2016 Clinical Learning Environment Review (CLER) site visit report, 88% of residents in group interviews reported following a standardized procedure for handling TOCs between shifts, and slightly more than half (53%) reported that faculty rarely supervised their shift-to-shift handoffs. During this visit, the CLER visitors reported that neither a standard mnemonic nor a written template was used during one observed handoff. In addition, resident responses to the annual WSUGME internal survey administered in fall 2016 revealed low rates of awareness (7% to 10%) of the specific TOC mnemonic required.

In this article, we detail our innovation for standardizing the use of the SAIF-IR mnemonic among primary care residents on inpatient services at our primary clinical site, Ascension Providence Rochester Hospital (APRH). Previous studies have confirmed the importance of providing residents with structured training about TOC mnemonic use via interventions such as bootcamps, didactic sessions, and/or simulated learning experiences, but little research is available on TOC postintervention reinforcement via direct observation of actual patient encounters.^12,13^

Our expectation was that use of a direct observation instrument, Transitions of Care-Clinical Evaluation Exercise (TOC-CEX), by faculty as both a monitoring and education tool would increase awareness and adoption of TOCs as a practice habit and contribute to culture change in the clinical learning environment.

## METHODS

On April 3, 2020, the Wayne State University Institutional Review Board determined that this study (Protocol IRB-20-03-1951) was exempt from review.

Located in southeast Michigan in the Metropolitan Detroit area, WSUGME provides administrative and curricular oversight of 7 residency programs with approximately 145 residents and 6 major hospital partners—the largest participating site being APRH, a 220-bed community hospital located in Rochester, Michigan, a suburb of Detroit. The study population included residents in 3 programs at APRH: internal medicine, family medicine, and transitional year. Using the TOC-CEX, faculty generated 223 observations of 95 residents, which took place at APRH between July 2017 and September 2019. During the 3 years studied, 138 unique residents were in the 3 programs, 96 of whom were interns and 42 of whom were postgraduate year (PGY) 2 and PGY 3 residents. A majority (86%) of the TOC-CEX observations were of interns, with the remainder (14%) being observations of PGY 2s and PGY 3s. Thus, the study sample reflects ratings of 99% of all interns during the study period but only 69% of possible residents.

WSUGME used 3 data sources to gauge the percent change in resident awareness and adoption of the mnemonic: (1) CLER site visit reports submitted by an ACGME team of evaluators in 2016 and in 2018; (2) responses to 2 questions about handoff use on an annual graduate medical education survey distributed to residents each October across 4 years (2016, 2017, 2018, and 2019); and (3) faculty observations of resident handoffs using the TOC-CEX from July 2017 through September 2019.

**Figure 1.**
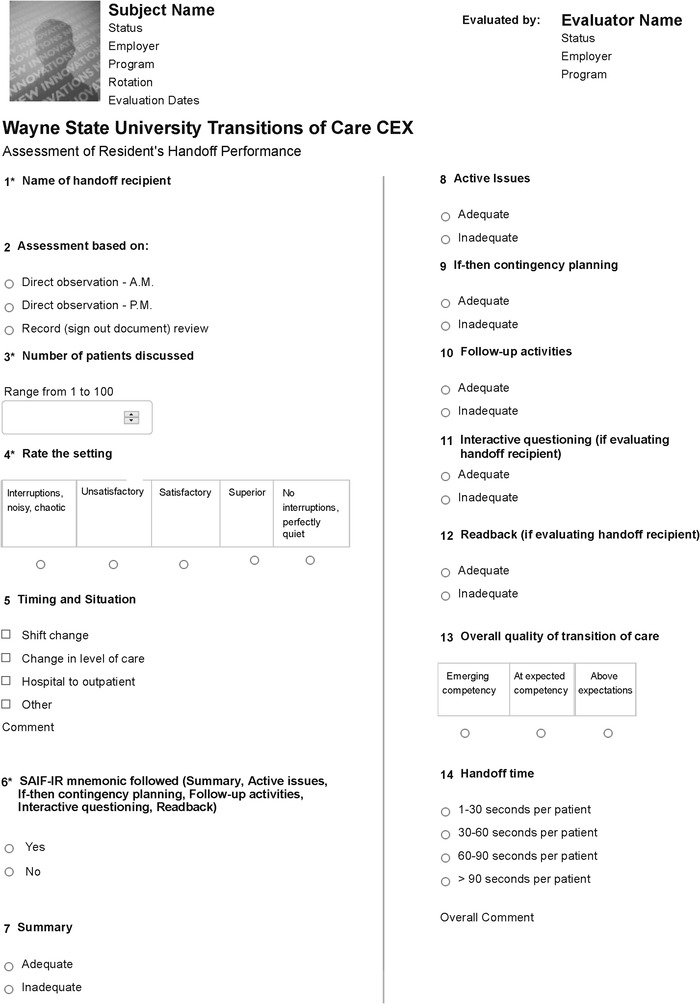
Wayne State University Transitions of Care-Clinical Evaluation Exercise direct observation tool.

WSUGME is dedicated to educating residents about the importance of a standardized TOC approach as a core practice habit in clinical settings. Training begins at the mandatory June orientation when all incoming interns are introduced to the SAIF-IR mnemonic. This mnemonic is reinforced later in the summer when all interns and PGY 2 residents are required to complete an objective simulated handoff evaluation training at the WSU medical school clinical skills center. The objective simulated handoff evaluation comprises a handoff exercise in pairs, with a PGY 2 rating a PGY 1's skill in using the SAIF-IR mnemonic by completing a shortened version of the TOC-CEX.^14^ Previous studies have found that simulation can be an effective method for teaching handoffs, particularly when accompanied by observer feedback and curricular requirements.^15^ Such deliberate practice allows residents to rehearse foundational skills across 2 yearly iterations in a low-stakes environment and also demonstrates the WSUGME commitment to policy monitoring while providing opportunities to refine handoff education.^11,16^ Beginning in summer 2017, WSUGME encouraged faculty adoption of the TOC-CEX as a direct observation tool (Figure 1).

The TOC-CEX was modeled after the Mini-Clinical Evaluation Exercise, part of a suite of clinical assessments recommended by the American Board of Internal Medicine.^17^ The tool asks basic information about who is handing off to whom, time of day, quality of setting, number of patients discussed, duration of handoff, whether the SAIF-IR mnemonic was used, an evaluation (adequate/inadequate) of each component of the mnemonic, and a numerical evaluation of the handoff as a whole (emerging competency/at expected competency/above expectations).

WSUGME makes the TOC-CEX available to faculty as an electronic template via New Innovations, a widely used web-based residency management system. The WSUGME director of education downloads the completed instruments. The administration protocol followed by APRH faculty varies slightly according to specialty. In internal medicine, 4 teams that include a senior resident and 2 interns meet twice daily (morning and afternoon) to conduct handoffs; faculty monitor the handoffs and conduct a written direct observation of each resident 3 or 4 times per month. In family medicine and transitional year, the rounding faculty member that week observes a daily sign-out in the afternoon between senior residents and interns, selecting 1 resident per day, typically the resident who has seen the most patients, so that by the end of the week, all residents have been assessed using the TOC-CEX. As the family medicine associate program director noted, “We stick to a schedule and make the process very familiar to all residents from the start of their training, setting expectations” about the required mnemonic on which they will be assessed every week, an approach “which has decreased resistance.” The internal medicine program director commented, “Faculty oversight of the handoff process by direct observation using the SAIF-IR TOC-CEX ensures that residents are competent in communicating with team members in the handoff process. We [also] do multiple rehearsals” as a regular component of clinical training. As a result, “Most of our residents agree that medical errors could be reduced by ensuring that patient information on the handoff report is up to date and by conducting sign-outs in a more systematic manner.” These comments suggest how the TOC-CEX as both an instructional tool and monitored practice has become embedded in APRH organizational culture as a necessary and expected component of training and central to quality care, rather than just another administrative burden.^18^

## RESULTS

Analysis of the 3 data sources confirmed our expectation that faculty use of a direct observation instrument in the clinical learning environment during 2017-2019 would increase awareness and adoption of the SAIF-IR mnemonic among residents. By the time of the 2018 CLER site visit, 100% of APRH residents in group interviews reported using a standardized TOC, with 86% using a written template. CLER site visitors also observed appropriate SAIF-IR mnemonic use together with faculty supervision during 2 observed handoffs. Further, APRH percentages reporting faculty supervision are well above national norms. According to the 2018 CLER National Report, when asked how frequently attending physicians supervise their shift-to-shift handoff process, 23.3% of respondents nationwide said daily and approximately 15% said weekly, compared with 29% of APRH residents reporting daily and 57% weekly supervision.^19^

**Figure 2.**
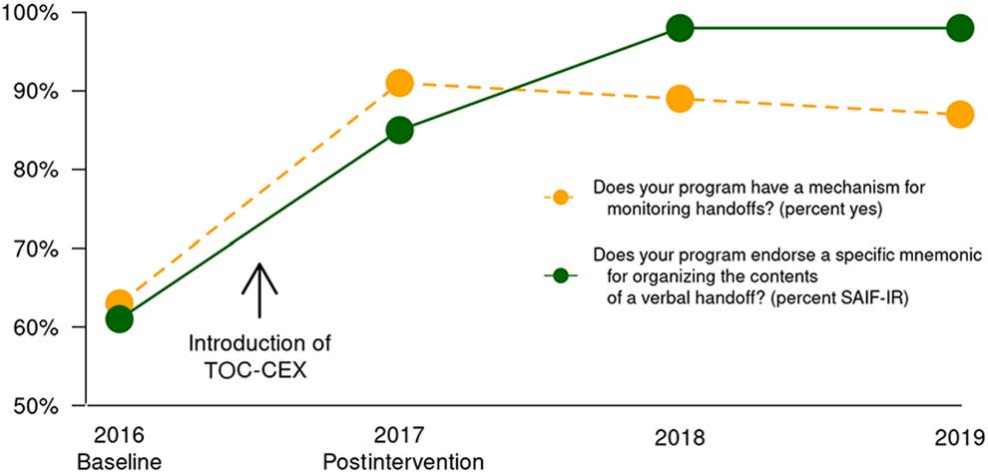
**Increases in Ascension Providence Rochester Hospital residents’ awareness of handoff mechanism and required mnemonic from 2016-2019. The dashed line represents the percentage of yes responses to question 1, “Does your program have a mechanism for monitoring handoffs?” The solid line represents the percentage correctly identifying the SAIF-IR mnemonic (summary, active issues, if-then contingency planning, follow-up activities, interactive questioning, readback) for question 2, “Does your program endorse a specific mnemonic for organizing the contents of a verbal handoff?”** Source: Graduate medical education resident surveys 2016-2019. TOC-CEX, Transitions of Care-Clinical Evaluation Exercise.

Most importantly, resident responses to questions about handoff use in the WSUGME internal survey in October of each academic year revealed significant increases in awareness of handoff monitoring across the study period (Figure 2). A *z*-test of equal proportions showed a significant rise in the percentage reporting yes for question 1, “Does your program have a mechanism for monitoring handoffs?” (*χ^2^*[3]=23.6, *P*<0.0001) and in the percentage identifying SAIF-IR in response to question 2, “Does your program endorse a specific mnemonic for organizing the contents of a verbal handoff?” (*χ^2^*[3]=45.0, *P*<0.0001). The change is attributed to the increase from 2016, prior to TOC-CEX introduction, to 2017, after TOC-CEX implementation (question 1: *χ^2^*[1]=12.4, *P*<0.0005; question 2: *χ^2^*[1]=10.1, *P*<0.0025). No other year-to-year changes were significant.

With regard to adoption of the required mnemonic for handoffs, faculty reported that the SAIF-IR mnemonic was used in 218 of 223 observations (98%). With regard to the overall quality of the handoffs, faculty rated 7 of 219 (3%) as emerging competency*,* 181 of 219 (83%) as expected competency, and 31 of 219 (14%) as above expectations. Four TOC-CEXs were returned with missing data. WSUGME found that the overall quality ratings showed very high interrater reliability; the intraclass correlation coefficient (ICC) of mean ratings was ICC[2]=0.96. This result indicates strong interrater agreement about the quality of a TOC.

## DISCUSSION

Adoption of the SAIF-IR mnemonic has been the core of TOC training in all our residency programs since 2012, but implementation of the TOC-CEX for faculty monitoring of resident use in the hospital site appears crucial. Via systematic participation in embedded clinical practices, residents observe faculty engagement in quality and safety, which is important because, as Myers and Nash argue, residents can become a “powerful force for changing hospital culture given their…natural ability to influence one another through social networks [and have] the potential to affect patient care outcomes through their knowledge, skills, and attitudes.”^18^ Implementation of the TOC-CEX proved both feasible and well accepted by residents, faculty, and the APRH chief medical officer, as the new practice was incorporated with minimal or no disruption in patient care and reflected an ethic of collaboration and teamwork in service of patient safety while also reinforcing a commitment to policy adherence. Further, adoption of the TOC-CEX is part of WSUGME's continuous quality improvement approach mandated by the ACGME, a model that focuses on innovation and growth by promoting management tools such as performance dashboards and SMART-formatted (specific, measurable, accountable, realistic, timely) goals to transform the culture of GME. A core continuous quality improvement principle is that improvement requires change that must be monitored.^20^

In terms of feasibility, WSUGME was able to address incoming interns’ lack of TOC preparedness without adding either a training burden (new residents are required to attend both the GME orientation and the objective simulated handoff evaluation) or substantial expenditure (we innovated the TOC-CEX in house and made it available electronically, at no cost, to residency faculty). With respect to training resources, WSUGME has access to a clinical skills center affiliated with our medical school that charges a fee for conducting the objective simulated handoff evaluations. However, any residency program could hold a similar TOC workshop at lower cost because no simulated patients are required. As explained earlier, in our training model, PGY 2s rate the skill of PGY 1s in using the SAIF-IR mnemonic during the objective simulated handoff evaluation.

Limitations of this study include implementation in a single setting (a community hospital with predominantly suburban patients) and a small study population of internal medicine, family medicine, and transitional year residents across 3 years. In addition, adoption of a direct observation instrument such as the TOC-CEX by specialties like dermatology might be less suitable because physicians in these domains do not regularly engage in acute hospital-based patient care and focus principally on hospital consulting activity. At the same time, the instrument is sufficiently modifiable so that medical educators can adapt it for implementation in a variety of other settings.

Future studies could address the piloting of a TOC-CEX instrument or innovation of a similar monitoring rubric for adoption in specialties other than primary care, perhaps in tandem with simulated exercises such as the objective simulated handoff evaluation, and compare outcomes across specialties. In addition, researchers might study TOC-CEX implementation by larger residency programs in other types of clinical learning environments, perhaps an urban hospital with a high patient volume and acuity. Although we describe one approach to reducing possible resident resistance to handoff monitoring, the exploration of other strategies (such as engaging residents in quality improvement projects addressing the enhancement of TOCs in their clinical setting) warrants investigation. Finally, WSUGME agrees with Rosenbluth that in subsequent TOC research, the “ideal data collection instrument would also include outcome measures…such as medical errors and adverse events, [which are] more difficult to document but also provide more valuable data about the impact of [TOC] curricula.”^21^ Such data will assist medical educators in determining the most effective handoff training approaches in the interest of optimizing patient safety.

## CONCLUSION

Faculty employment of the TOC-CEX in the clinical setting appears key to ensuring adoption by residents programwide. As a result, WSUGME has been able to standardize the SAIF-IR mnemonic as an integral component of the primary care programs at our main teaching hospital.

## Notes

*This article meets the Accreditation Council for Graduate Medical Education and the American Board of Medical Specialties Maintenance of Certification competencies for Patient Care, Medical Knowledge, Systems-Based Practice, and Practice-Based Learning and Improvement.*
